# Psychometric evaluation of the Urdu version of Inventory of Callous-Unemotional Traits: A multi-phase validation

**DOI:** 10.1371/journal.pone.0353300

**Published:** 2026-07-09

**Authors:** Sania Mazher, Sobia Masood, Takashi Arai

**Affiliations:** 1 Graduate School of Arts and Letters, Psychology Department, Tohoku University, Sendai, Japan; 2 Psychology Department, Rawalpindi Women University, Rawalpindi, Pakistan; Port Said University Faculty of Nursing, EGYPT

## Abstract

Callous-unemotional (CU) traits are central to the specifier with Limited Prosocial Emotions in the DSM-5 and ICD-11, reflecting severe emotional and behavioral disturbances. Despite the global use of the Inventory of Callous-Unemotional Traits (ICU), no validated version existed in Urdu. The aim of the present study was to translate, culturally adapt, and validate the Urdu version of the Inventory of Callous-Unemotional Traits (ICU-U) among Pakistani youth. The study consisted of three phases: translation and cultural adaptation of the instrument, assessment of test-retest reliability, and evaluation of structural validity through confirmatory factor analysis, alongside testing of convergent and discriminant validity. Test-retest reliability with bilingual youth (N = 60) was excellent, with the Urdu–Urdu condition demonstrating the highest stability. CFA conducted on a sample of 300 young adults (aged 18–25) supported a refined 12-item two-factor model (Callousness and Uncaring) over the original three-factor and bifactor models, aligning with previous international findings. Internal consistency for both subscales was satisfactory, and the ICU-U demonstrated strong convergent and discriminant validity through expected associations with criminal sentiments and self-control measures. The findings establish the ICU-U as a culturally relevant and psychometrically sound instrument for identifying CU traits in Urdu-speaking populations. Its use holds promise in clinical, educational, and forensic settings for early detection and intervention planning.

## Introduction

The most recent editions of major diagnostic systems, including the Diagnostic and Statistical Manual of Mental Disorders, Fifth Edition [1] and the International Classification of Disease, 11th Revision [2], have included the specifier with Limited Prosocial Emotions to describe a subgroup of young people with severe behavioral issues who have high levels of CU traits. CU characteristics include a shallow or limited affect, lack of empathy, lack of regret, and a lack of concern for one’s success in important tasks, according to Frick [3].

According to theory, CU traits are an affective aspect in children [4] and the larger construct of psychopathy in adults [4]. Additionally, studies showing links between CU characteristics and especially serious (i.e., aggressive) and persistent types of antisocial behaviour supported their inclusion in the clinical classification [3].

It has been demonstrated, for instance, that young people with higher CU features use weapons more frequently [4,5] and exhibit more destructive types of violence [6–9]. Furthermore, even when there are no significant conduct issues, youth with higher CU features are observed to have more psychosocial and relationship dysfunction [10–12]. For instance, it has been demonstrated that young people with higher CU traits face more rejection from their peers and have a greater likelihood of being labelled as “mean” by them [13–15]. Moreover, CU characteristics appear to indicate an etiologically unique group of young people with serious behavioural issues who exhibit less sensitivity to punishment and less emotional responsiveness to the pain of others [16].

According to [17], however, it seems that roughly 25% to 30% of young adolescents with serious conduct issues had elevated levels of CU characteristics. Thus, it’s critical to evaluate these characteristics in a valid and reliable way. Despite their size, this subset of children and teens needs to be evaluated and treated professionally for a number of crucial reasons, such as the fact that their behavioural issues are more serious and persistent. In this instance, they also exhibit an increased level of violence, which is more goal-oriented—for dominating the other individual or for personal gain—and tends to injure the victims more [18,19]

The Psychopathy Check List-Youth Version (PCL-YV) [20] and the Antisocial Process Screening Device (APSD) [21] are two frequently used tools in research due to the significance of evaluating individuals for their CU features, especially with conduct problems. The PCL-YV’s drawback is the amount of time required to complete the assessment; even highly qualified professionals need about 60–90 minutes. As for the APSD, it is difficult to verify the significant aspects of CU characteristics because only six of the 20 items on the APSD assess CU characteristics, which can be self-reported by young people, their guardians, or their instructors [21]. The inventory of CU traits was developed by Frick [22] to expand the CU characteristics items on the APSD in response to these shortcomings of other assessments. It has 24 items with 4-point Likert scale codes. Versions of the Youth Self-Report, Parent Report, Teacher Report, and Parent Report (Preschool) are available. Callousness, which describes actions that include a lack of empathy, guilt, and regret; Uncaring, which denotes a lack of concern for one’s performance on tasks and for the feelings of others; and Unemotional, which denotes a lack of emotional expression, are the three main characteristics of CU that have been found in studies [23].

Various samples with varying translations have usually supported these three characteristics [24–26]. For instance, these three factors were discovered for the first time by Essau et al. [23] using an exploratory factor analysis in a sample of 1443 Germans aged 13–18 from the general population. They also discovered a common general characteristic that included all of the items (i.e., the bifactor model) of the self-report version, and the factor structure was the same for both boys and girls. Using the self-report version, Kimonis and colleagues [22] validated this factor structure in a sample of 248 American juvenile offenders aged 12–20. The Unemotional factor’s internal consistency was low (0.53), and two Callousness items (items 2 and 10) were eliminated from their model because of poor item-total correlations. The bifactor model was validated using a self-report version in 540 Italian children (mean age = 12.7 years) by Ciucci and colleagues [12], and the factor structure was consistent across grade and sex.

A few recent studies have questioned the validity of scales and the bifactor model [27–29]. Willoughby and colleagues [29] examined the parent ratings of the ICU in 1078 American first-graders (mean age = 7.3 years) and discovered that the best fit was a two-factor model that distinguished callous-unemotional (including nine Callousness and two Unemotional items from the original scale) and empathic-prosocial (including eight Uncaring, two Callousness, and three Unemotional items) factors. Furthermore, not a single item was cross-loaded on more than one variable.

In a study of 250 American teenagers with significant conduct problems (ages 6–12), Hawes and colleagues [28] developed a structure based on two factors (callousness and uncaring) using 12 of the original 24 items of the parent-report version. In a research by Houghton and colleagues [30] that examined the factor composition of self-report ICU in 268 Australian children aged 7–12, a two-factor model worked better than a three-factor model. The two-factor model’s unemotional items were all removed due to low internal reliability and model fit. Each of the eight items in the Callousness and Uncaring categories exhibited internal reliabilities of 0.77 and 0.85, respectively, suggesting sufficient reliability. Eight pairs of error terms were connected in order to improve model fit even more.

Using 613 Chinese males who were detained among the Chinese samples, Zhang and colleagues [31] investigated the factor structure and psychometric properties of the Chinese equivalent of the ICU. According to the findings of the confirmatory factor analysis, the three-factor model that included 24 items from the original ICU scale did not show a good match to the data. However, the two-factor with factors of callousness and uncaring—which included only eleven items—showed the most correlation with the data. The Youth Psychopathic Traits Inventory and the Antisocial Process Screening Device were also compared with the two-factor ICU in the study, and both instruments showed comparable and noteworthy correlations with the relevant criteria of violence, psychopathy, and compassion.

In a different study, Allen and colleagues [32] evaluated the invariance between the Chinese and British populations using both the original 24 items and the shorter ICU (11 items). The study comprised 364 Chinese children, aged 10–13, who were studying in schools in Guangdong, China, and 437 British students, aged 11–14, from the East of England. Both the original 24-item and the 11-item ICUs showed strong internal consistency for the sub-factor and overall scores, but not for the unemotional factor in either sample. The unemotional measure has demonstrated low internal consistency and poor construct validity in previous research, per a recent meta-analysis study [33].

Although the ICU has been widely validated across different cultural contexts, findings regarding its factor structure remain inconsistent. While early studies generally supported the original three-factor or bifactor structure, more recent research has increasingly questioned the reliability and distinctiveness of the Unemotional dimension. Several cross-cultural studies have instead reported improved fit for shortened two-factor models consisting primarily of Callousness and Uncaring dimensions. These inconsistencies suggest that the underlying structure of CU traits may vary across linguistic and cultural contexts, as cultural norms surrounding emotional expression and interpersonal behavior may influence how CU traits are interpreted and reported across populations [34,35], highlighting the need for further validation studies in underrepresented populations such as Urdu-speaking youth.

Therefore, the present study contributes not only to the linguistic adaptation of the ICU into Urdu but also to the broader theoretical discussion regarding the cross-cultural stability of CU trait structures. By comparing competing factor models within a Pakistani sample, the study aims to clarify whether previously reported two-factor solutions generalize to Urdu-speaking populations. Despite the widespread international adaptation and validation of the ICU, no validated Urdu version currently exists. The precise diagnosis and treatment of conduct disorder and psychopathic features in Urdu-speaking populations, especially among Pakistani adolescents and young adults, is severely restricted by this shortcoming. Therefore, the present study aimed to translate, culturally adapt, and examine the factor structure and psychometric properties of the Urdu version of the Inventory of Callous-Unemotional Traits (ICU-U) in a Pakistani sample.

The construct validity of the ICU has been supported by numerous studies that show a strong correlation between CU traits and violent, delinquent, and antisocial conduct [18,22,36]. Nevertheless, no research has been done to examine the convergent and discriminant validity of the ICU in Urdu. By testing the Urdu version’s convergent and discriminant validity, we address this gap.

The primary objective of the research was to examine the psychometric qualities of the self-report ICU in young people selected from the community. In particular, (1) we will use a series of CFAs to compare competing measurement models previously examined in the literature, including an intercorrelated three-factor model, a three-factor bifactor model, and a two-factor model, and (2) we will examine the scale’s convergent and discriminant validity. We anticipated that this sample would duplicate the two-factor structure (Callousness and Uncaring) based on previous research [37].

## Method

There were three phases in this study. A multi-phase design was adopted to ensure comprehensive cross-cultural validation of the ICU-U. The first phase focused on linguistic and cultural adaptation of the instrument, the second phase examined temporal stability across language conditions, and the third phase evaluated the structural and construct validity of the translated scale. This sequential approach is consistent with recommendations for psychometric validation studies and allowed the study to examine multiple aspects of reliability and validity within the Urdu-speaking context. The details of the three phases are below.

### Phase I: Translation of inventory of callous unemotional traits

The initial phase’s goal was to use forward and backward translation techniques to obtain the Urdu translation of ICU from English [38]. To obtain a conceptually equivalent Urdu version of ICU, phase 1 was carried out. Instead of only linguistic or literary equality, the focus was on cross-cultural and cultural equality. The following is the instrument detail:

#### Instrument.

The original ICU [39] comprises 24 items with three subscales: callousness, uncaring, and unemotional. A 4-point Likert scale, ranging from Definitely true (3) to Not at all true (0), serves as the basis for the response structure. Frick [39] reports moderately high (Cronbach alpha = .81) internal consistency of ICU (alpha values for the subscales ranged from  .53 to  .81).

#### Inclusivity in global research.

Additional information regarding the ethical, cultural, and scientific considerations specific to inclusivity in global research is provided in the Supporting Information (S1 Checklist).

#### Procedure.

We translated ICU using the forward-backward translation approach in accordance with Brislin’s [38] recommendations. The original author gave permission for the translation and validation of the ICU. Five bilinguals were assigned to translate the ICU into Urdu while maintaining conceptual equivalence and cultural relevance. Three psychometric experts took part in a committee review of the five Urdu translations of ICU that were received, and the best translations were chosen. Particular attention was given to ensuring that the translated items were culturally understandable, age-appropriate, and conceptually relevant for Urdu-speaking Pakistani youth. During the expert review process, minor wording modifications were made to improve clarity and cultural comprehensibility while preserving the original meaning of the items. The chosen questions were then back-translated into the original language, which is English. The English (Original English and back-translated English) scale versions were then examined and assessed to make linguistic and cultural adaptations in order to guarantee that the scale’s meaning was maintained.

### Phase II: Test-retest reliability of ICU-U

Phase II involved the test-retest reliability of the translated ICU. The test-retest reliability of the instrument’s translated Urdu and English versions was assessed during this step.

#### Sample.

From Islamabad Model College for Boys, sixty male youths were chosen. The college sample, which at the time consisted exclusively of male students, was selected based on accessibility, participant availability, and the requirement for bilingual respondents fluent in both Urdu and English for cross-language validation purposes. Although the use of a male-only sample may introduce potential gender bias and limit the generalizability of the temporal stability findings to female populations, the test-retest phase was intended as a preliminary assessment of cross-language reliability rather than population-level validation. The age range of the sample was 18–20 years old. The participants were bilingual and fluent in both Urdu and English.

#### Inclusivity in global research.

Additional information regarding the ethical, cultural, and scientific considerations specific to inclusivity in global research is provided in the Supporting Information (S1 Checklist).

#### Procedure.

Participants who were readily available and ready to take part were given the ICU questionnaire after written informed consent was obtained. Ethical approval for the study was granted by the Institutional Review Board of the National Institute of Psychology, Quaid-i-Azam University, Islamabad. The data was collected using paper and pencil survey from 10^th^ January 2020–30^th^ April 2020. Prior to participation, all individuals were informed about the study’s purpose, procedures, and their rights, including the voluntary nature of participation and the assurance of anonymity. Written informed consent was obtained from each participant before completing the survey. Participants signed a consent form that was stored separately from the questionnaires to ensure confidentiality. After that, the data was entered into the secured electronic database, which was accessed for the research purpose on 19^th^ August, 2025. All survey responses were anonymous, and participants were not asked to provide identifying information. The authors did not have access to any information that could identify individual participants during or after data collection. Participants were given instrument booklets, and they were asked to fill them out truthfully.

The sample was split into four equal groups of 15 participants each (total = 60), with accordance of previous research [40]. The assessment was administered in such a way that, in the *English–Urdu* test–retest condition, the participants (*n* = 15) were given the original version of ICU, and two weeks later, the same participants were given the instrument again using the Urdu translation. The original version (English) of the instrument was given to the participants (*n* = 15) in the *English–English* test–retest condition, and two weeks later, the same participants were given the exact same questionnaire again. Participants (*n* = 15) were given the translated version of the instrument in the *Urdu–English* test–retest condition. The original form was then given to the same respondents as a follow-up test. Lastly, in the fourth condition, *Urdu–Urdu* test–retest, participants (*n* = 15) were given the Urdu-translated version of the instrument, and the same subjects were retested two weeks later.

#### Statistical analysis.

A test-retest was conducted using JASP version 0.19. Temporal stability of the ICU-U was examined over a two-week interval. Pearson correlations (r) and Intraclass Correlation Coefficients (ICC; two-way mixed-effects, absolute agreement) were calculated to assess score stability across administrations.

### Phase III: Psychometric properties of ICU-U

Phase III was conducted in order to analyze the translated ICU’s factor structure and convergent and discriminant validity.

#### Sample.

Three hundred young adults from various universities, both public and private, in Rawalpindi and Islamabad were included in the sample (179 men and 121 females). Respondents were between the ages of 18 and 25 (*M* = 19.50, *SD* = 1.11 years). Convenience sampling was used to approach the university sample due to feasibility and accessibility considerations during data collection. Although this approach facilitated recruitment from multiple universities, it may limit the generalizability of findings to broader Pakistani populations.

#### Instruments.

CFA was done on the ICU-U. The details of the instrument are given in Phase I (see Instrument). Convergent validity was evaluated using the Criminal Sentiments Scale-Modified [CSS-M; 41], which was modified and translated into Urdu [42]. This measure is divided into three sub-scales: Identification with Criminal Others, Tolerance for Law Violations, and Law, Court, and Police. With total scores ranging from 0–82, all 41 items on the test are scored on a 3-point Likert scale from *Disagree* (0) to *Agree* (2). Higher scores reflect higher levels of criminal attitudes and are suitable to express convergent validity with ICU-U; CSS-M [Cronbach alpha = .97, 42] should positively correlate with ICU-U because high CU traits are linked with high criminal attitudes theoretically and empirically. A brief Self-Control Scale adapted in Urdu [43] was used to measure discriminant validity. This scale consisted of 13 items, where each item is measured on a 5-point Likert scale from *not at all like me* (1) to *very much like me* (5) with relatively high reliability [Cronbach alpha = .85, 44]. Psychopathy and callous-unemotional traits are associated with impulsivity and poor self-regulation, so individuals with higher CU traits typically have lower self-control, leading to a negative correlation between the self-control scale and ICU-U.

#### Inclusivity in global research.

Additional information regarding the ethical, cultural, and scientific considerations specific to inclusivity in global research is provided in the Supporting Information (S1 Checklist).

#### Procedure.

Participants who were readily available and ready to take part were given the questionnaires after written informed consent was obtained. Ethical approval for the study was granted by the Institutional Review Board of the National Institute of Psychology, Quaid-i-Azam University, Islamabad. The data was collected using a paper and pencil survey from 10^th^ January 2020–30^th^ April 2020. Prior to participation, all individuals were informed about the study’s purpose, procedures, and their rights, including the voluntary nature of participation and the assurance of anonymity. Written informed consent was obtained from each participant before completing the survey. Participants signed a consent form that was stored separately from the questionnaires to ensure confidentiality. After that, the data was entered into the secured electronic database, which was accessed for the research purpose on 19^th^ August, 2025. All survey responses were anonymous, and participants were not asked to provide identifying information. The authors did not have access to any information that could identify individual participants during or after data collection. The researcher thanked them when they completed the questionnaire.

#### Statistical analysis.

CFA was conducted using JASP version 0.19 in order to validate the factor structure of ICU on the adult population of Pakistan. To assess model fit, we took into account the RMSEA, goodness-of-fit index (GFI), IFI, and CFI. Accordingly, IFI and CFI values greater than. 90 frequently signifies a good and satisfactory fit to the data. For the RMSEA, values smaller than. 08 denotes an acceptable and good fit to the data, respectively. Convergent and discriminant validity was assessed by calculating Pearson correlations between ICU-U, CSS-M, and the Brief Self-Control Scale.

## Results

The results of all phases are described below.

### Results of translation and cultural adaptation

The back-translated English version of the ICU was reviewed by the original scale author, who confirmed that all items retained their original meaning and intent. No ambiguities, contradictions, or conceptual inconsistencies were identified between the original and back-translated versions. As a result, the Urdu version of the ICU was judged to demonstrate adequate linguistic and conceptual equivalence and was considered suitable for cross-cultural validation and field testing.

### Results of test-retest reliability

The test-retest reliabilities of the translated (Urdu) version of the study instrument were established using the data gathered from young people using the previously described approach. The following are the results that were obtained.

All four groups have positive test-retest reliability and statistical significance, as shown in Table 1. In accordance with the criteria for test-retest reliability set by Sparrow and colleagues [45], good to excellent test-retest reliability is indicated by Pearson correlations and intraclass correlation coefficients (ICC; absolute agreement) above  .53 (<.40 = poor,  .40 –  .59 = fair,  .60 –  .74 = good, and  .75–1.00 = excellent). However, given the small sample size in each retest group (*n* = 15), these findings should be interpreted as preliminary evidence of temporal stability. Since ratings remain constant over time, these findings confirmed that they possessed external consistency and temporal validity within the sample. The results also showed that, of the four groups, the Urdu–Urdu group’s correlation coefficients for the scale were stronger and higher than the English–English group’s. This suggests that, in comparison to the English version, the translated Urdu form was found to have greater understanding. These results demonstrated that young male Pakistani people were able to validate the translated version of the ICU across languages.

**Table 1:** Test–Retest Reliabilities of English and Urdu Version of the Inventory of Callous Unemotional Traits (*N* = 60).

Scales	*n*	*r*	*ICC*
English-English	15	.82^**^	.53
English-Urdu	15	.53^**^	.53
Urdu-Urdu	15	.85^**^	.83
Urdu-English	15	.73^**^	.84
Overall	60	.73	.68

** *p* < .01.

### Results of psychometric properties

#### Confirmatory factor analysis.

Table 2 shows the fit indices of competitive models used in the current study. Fit indices showed an unacceptable fit for the inter-correlated three-factor model (M1; *χ²* = 1255.82, *df* = 249, CFI = 0.77, TLI = 0.74, RMSEA = 0.12) and for the original three-factor bifactor (M2; *χ²* = 1255.81, *df* = 249, CFI = 0.76, TLI = 0.74, RMSEA = 0.11). The two-factor model of the ICU 12 had significantly better fit than the M1 or M2, but the fit indices were still unsatisfactory (M3; CFI = 0.89, TLI = 0.86, RMSEA = 0.08). After adding error variance, the values of the GFI, IFI, CFI, χ²(df), and RMSEA fell into the acceptable range. A good fit was indicated in Model 3 (adjusted) of ICU 12.

**Table 2:** Goodness-of-fit Indices for the Different Models of ICU-U (*N* = 300).

Model	*χ² (df)*	TLI	IFI	CFI	RMSEA	SRMR	BIC
M1 (Inter-correlated three factor)	1255.82^***^ (249)	.74	.77	.77	.12	.08	14671.37
M2 (three factor bi-factor)	1255.81^***^ (249)	.74	.76	.76	.11	.07	14671.37
M3 (Two-factor)	180.19^***^ (53)	.86	.89	.89	.08	.06	6952.94
M3 (Adjusted)	102.98^***^ (45)	.92	.95	.95	.06	.04	6921.37

*Note. χ² =* Chi-Square; *df* = Degree of Freedom; TLI = Tucker-Lewis Index; IFI = Incremental Fit Index; CFI = Comparative Fit Index; RMSEA = Root Mean Square Error of Approximation; SRMR = Standardized Root Mean Square Residual; BIC = Bayesian.

In a two-factor model, the factor loading for the ICU-U was displayed in Table 3. Every item displayed factor loading values above the cutoff point (.30).  .41 to  .73 is the range of factor loadings for the two-factor model.

**Table 3:** Factor Loadings for the Two-factor Model for ICU-U 12 (*N* = 300).

Item	Callousness	Uncaring
(4) I do not care who I hurt to get what I want	.43	
(6) I do not show my emotions to others	.63	
(9) I do not care if I get into trouble	.72	
(11) I do not care about doing things well	.65	
(12) I seem very cold and uncaring to others	.54	
(18) I do not feel remorseful when I do something wrong	.48	
(21) The feelings of others are unimportant to me	.41	
α (ω)	.76 (.73)	
(5)I feel bad or guilty when I do something wrong (R)		.67
(8)I am concerned about the feelings of others (R)		.73
(16)I apologize to persons I hurt (R)		.68
(17)I try not to hurt others’ feelings (R)		.68
(24)I do things to make others feel good (R)		.69
α (ω)		.82 (.83)

#### Convergent and discriminant validity.

Convergent and Discriminant validity were conducted using JASP version 0.19. The analysis of the results revealed ICU-U was positively (*r* = .84, *p* < .01) related to CSS-M and negatively (*r* = −.84, *p* < .01) with the Brief Self-Control Scale. Results indicate that CU traits are related to greater criminal attitudes, which attests to convergent validity. Similarly, CU traits and self-control are strongly and negatively correlated, which indicates that greater CU traits are related to less self-control, which attests to discriminant validity.

## Discussion

The current study aimed to translate, culturally modify, and evaluate the ICU in Urdu among young people in Pakistan. The findings provide the ICU-U with excellent psychometric support, demonstrating its validity and reliability as a tool for evaluating callous-unemotional (CU) qualities in Urdu-speaking cultures. Given the lack of validated instruments in Urdu for identifying young people with Limited Prosocial Emotions and associated behavioral issues, this is a noteworthy contribution.

In Phase I, we translated ICU using the forward-backward translation approach in accordance with Brislin’s [38] recommendations. No ambiguities, contradictions, or conceptual inconsistencies were identified between the original and back-translated versions. As a result, the Urdu version of the ICU was judged to demonstrate adequate linguistic and conceptual equivalence and was considered suitable.

Test-retest results were favorable. All test-retest groups showed strong reliability (ICC values between  .53 and  .84), with the Urdu–Urdu condition showing the strongest reliability (r = .85; ICC = .83). This shows not only strong temporal stability but also that the Urdu version may provide greater clarity and cultural resonance for native speakers compared to the original English version. These results are consistent with cross-national research highlighting the importance of culturally appropriate instruments [38,40].

Results of the CFA indicated that the first proposed inter-correlated three-factor and bi-factor models did not fit the data in the Pakistani sample [3,22]. Instead, a more nuanced two-factor model including Callousness and Uncaring fitted better, consistent with recent findings in several cultural contexts [28–30]. This finding may indicate that the affective and interpersonal dimensions of CU traits are more readily identifiable and culturally interpretable within collectivistic contexts such as Pakistan, whereas unemotionality may be expressed differently across cultures. These findings support growing evidence that the structure of CU traits may not be universally stable across cultural contexts and highlight the importance of culturally sensitive psychometric validation.

Our model had 12 items, with all factor loadings above the minimum threshold of 0.30, while these only had 11 items and a two-factor model [28–30]. We wanted to ensure that the factor structure of the ICU was adequate for the local population [31]. There are occasions when specific subscales and items have either been changed or removed in studies as a function of their psychometric performance; this includes modifications so that the scale is more appropriate for the underlying population. This methodological decision is consistent with previous cross-cultural psychometric validation studies which have deleted some items or subscales based on a lack of relevance or lower psychometric properties.

The internal consistency of the two factors was satisfactory (α = .76 for Callousness and α = .82 for Uncaring), showing that the ICU-U reliably measures these two factors of CU. The poor fit and low reliability of the Unemotional sub-scale, consistent with prior research [32–33], led to its removal from the final model. This supports the recent consensus that unemotionality may not be psychometrically distinct or consistently measurable across cultures and age groups.

The ICU-U also demonstrated excellent convergent validity by showing a strong positive relation with the Criminal Sentiments Scale – Modified (r = .84, p < .01), confirming that CU characteristics are related to pro-criminal attitudes. This supports previous results that CU characteristics predict antisocial and aggressive behaviors [18,19,22]. From a theoretical perspective, these findings align with developmental psychopathology models suggesting that CU traits are associated with reduced emotional responsiveness, impaired empathy, and diminished behavioral regulation, all of which contribute to antisocial attitudes and self-regulatory difficulties [19,22]. The strong associations observed in the present study further support the construct validity of the ICU-U within the Pakistani cultural context.

Moreover, the ICU-U showed strong discriminant validity, as shown by a significant negative correlation with the Brief Self-Control Scale (r = –.84, p < .01). This result is theoretically sound, as lower self-control is a known correlate of CU characteristics and psychopathy [43,44]. These findings enhance confidence in the scale’s ability to differentiate CU traits from other, distinct psychological constructs.

Beyond providing a translated instrument, the present study contributes to the broader literature on CU traits by demonstrating that psychometric structures established primarily in Western populations may not fully generalize across cultural and linguistic settings. The findings, therefore, emphasize the importance of examining the cultural variability of CU trait expression and measurement.

### Implications

An important gap in the evaluation of CU traits available for clinical, educational, and forensic contexts in Pakistan is filled by the availability of a validated ICU in Urdu. Because of their strong and significant relationship with aggressive behaviors and persistent conduct problems, early identification of CU characteristics is crucial [3,6]. In addition to practical contexts like schools, juvenile justice systems, and mental health institutes, ICU-U can be an important tool for research.

Furthermore, the simplified two-factor model improves clinical usage and interpretability by distinctly differentiating Callousness and Uncaring characteristics, which are observable and measurable in real-world contexts. This clarity facilitates psychologists and practitioners to better recognize specific emotional and motivational problems in youth who are at-risk. For instance, interventions can be developed to enhance empathy and emotional responsiveness in those who are high on Callousness, while interventions targeting responsibility and engagement may be more efficient for those who exhibit uncaring characteristics. Thus, this model not only facilitates assessment but also guides the development of more focused and efficient treatment plans.

### Limitations and future directions

Despite encouraging results, the study has some limitations. One limitation of this study is the small subsample used to assess ICU-U test–retest reliability (n = 15), which may reduce the precision of the stability estimates. Furthermore, the use of convenience sampling from urban university and college populations may restrict generalizability to other age groups, socio-economic classes, or rural areas. Moreover, the gender distribution in cross-language validation was limited to male sample, which may overlook gender-specific patterns in CU trait expression. One limitation is that the study did not assess the ICU Urdu version’s predictive validity for real-world behaviors like school misconduct, aggression, or recidivism.

Future research should aim to validate the ICU-U among clinical and juvenile offender populations, assess gender invariance, and explore the predictive validity of the scale for real-world behavioral outcomes such as school misconduct, aggression, or recidivism. Additionally, longitudinal studies would help clarify the stability and developmental trajectory of CU traits in Pakistani youth.

## Conclusion

In summary, the Urdu version of the ICU-U demonstrates good psychometric properties, including preliminary evidence of temporal stability, a robust two-factor structure, high internal consistency, and strong convergent and discriminant validity. This makes the ICU-U a culturally sensitive and reliable questionnaire for evaluating CU traits in the Urdu-speaking sample, with wide implications for research, diagnosis, and intervention planning.

## Supporting information

S1 Checklist Inclusivity in global research questionnaire. Completed PLOS ONE questionnaire addressing inclusivity and ethical considerations in global research.
(DOCX)

S2 File Inventory of Callous–Unemotional Traits (English version). Original English version of the Inventory of Callous–Unemotional Traits.
(PDF)

S3 File Inventory of Callous–Unemotional Traits (Urdu version). Urdu-translated and culturally adapted version of the Inventory of Callous–Unemotional Traits (ICU-U).
(PDF)
