# Supplementary material for: Psychometric evaluation of the Urdu version of Inventory of Callous-Unemotional Traits: A multi-phase validation
Source: PLoS One. 2026 Jul 9;21(7):e0353300. doi: 10.1371/journal.pone.0353300 (PMC13349191; doi:10.1371/journal.pone.0353300)
Supplement: S2 File — Original English version of the Inventory of Callous–Unemotional Traits. (PDF) [file pone.0353300.s002.pdf]

ICU
(Youth Version)

Name: ____________________

Date Completed: ____________________

<u>Instructions:</u> Please read each statement and decide how well it describes you. Mark your answer by circling the appropriate number (0-3) for each statement. Do not leave any statement unrated.

	Not at all true	Somewhat true	Very true	Definitely True
1. I express my feelings openly.	0	1	2	3
2. What I think is "right" and "wrong" is different from what other people think.	0	1	2	3
3. I care about how well I do at school or work.	0	1	2	3
4. I do not care who I hurt to get what I want.	0	1	2	3
5. I feel bad or guilty when I do something wrong.	0	1	2	3
6. I do not show my emotions to others.	0	1	2	3
7. I do not care about being on time.	0	1	2	3
8. I am concerned about the feelings of others.	0	1	2	3
9. I do not care if I get into trouble.	0	1	2	3
10. I do not let my feelings control me.	0	1	2	3
11. I do not care about doing things well.	0	1	2	3
12. I seem very cold and uncaring to others.	0	1	2	3
13. I easily admit to being wrong.	0	1	2	3
14. It is easy for others to tell how I am feeling.	0	1	2	3
15. I always try my best.	0	1	2	3
16. I apologize ("say I am sorry") to persons I hurt.	0	1	2	3
17. I try not to hurt others' feelings.	0	1	2	3
18. I do not feel remorseful when I do something wrong.	0	1	2	3
19. I am very expressive and emotional.	0	1	2	3
20. I do not like to put the time into doing things well.	0	1	2	3

21. The feelings of others are unimportant to me.	0	1	2	3
22. I hide my feelings from others.	0	1	2	3
23. I work hard on everything I do.	0	1	2	3
24. I do things to make others feel good.	0	1	2	3

Unpublished rating scale by Paul J. Frick, Department of Psychology, University of New Orleans (pfrick@uno.edu) .
