# Supplementary material for: Psychometric evaluation of the Urdu version of Inventory of Callous-Unemotional Traits: A multi-phase validation
Source: PLoS One. 2026 Jul 9;21(7):e0353300. doi: 10.1371/journal.pone.0353300 (PMC13349191; doi:10.1371/journal.pone.0353300)
Supplement: S3 File — Urdu-translated and culturally adapted version of the Inventory of Callous–Unemotional Traits (ICU-U). (PDF) [file pone.0353300.s003.pdf]

Inventory of Callous Unemotional Traits

ہدایات: دیئے گئے بیانات کو غور سے پڑھیں اور اپنی شخصیت کو سامنے رکھتے ہوئے دیے گئے پیمانے کی مدد سے یہ بتائیں کہ یہ آپ کے بارے میں کس حد تک درست ہیں۔

نمبر شمار	بیانات	بالکل درست نہیں	کچھ حد تک درست	بہت حد تک درست	بالکل درست
1	میں اپنے احساسات کا برملا اظہار کرتی/کرتا ہوں۔				
2	میرے خیال کے مطابق جو درست اور غلط ہے، وہ دوسرے لوگوں کی سوچ سے مختلف ہے۔				
3	مجھے اس بات کی پرواہ ہوتی ہے کہ میرے سکول یا کام کی کارکردگی کتنی بہتر ہے۔				
4	مجھے اس بات کی پرواہ نہیں کہ میں جو چاہتی ہوں اسے حاصل کرنے کے لئے کسی کو تکلیف پہنچاؤں۔				
5	جب میں کچھ غلط کروں تو مجھے بُرا لگتا ہے اور احساسِ گناہ ہوتا ہے۔				
6	میں اپنے جذبات دوسروں پر ظاہر نہیں کرتی/کرتا۔				
7	میں وقت پر پہنچنے کی پرواہ نہیں کرتی/کرتا۔				
8	مجھے دوسروں کے احساسات کا خیال ہوتا ہے۔				
9	میں اگر مشکل میں پڑ جاؤں تو مجھے کوئی پرواہ نہیں ہوتی۔				
10	میں اپنے احساسات کو خود پر حاوی نہیں ہونے دیتی۔				
11	میں چیزیں بہتر طریقے سے کرنے کی پرواہ نہیں کرتی/کرتا۔				
12	میں دوسروں کو سرد مہر اور پرواہ نہ کرنے والی/والا لگتی/لگتا ہوں۔				
13	میں با آسانی اپنے غلط ہونے کو تسلیم کر لیتی/لیتا ہوں۔				
14	دوسروں کے لئے یہ بتانا آسان ہے کہ میں کیسا محسوس کر رہی/رہا ہوں۔				
15	میں ہمیشہ بہترین کوشش کرتی/کرتا ہوں۔				
16	میں جن لوگوں کو تکلیف پہنچاؤں ان سے معذرت کر لیتی/لیتا ہوں۔				
17	میری کوشش ہوتی ہے کہ دوسروں کے احساسات کو ٹھیس نہ پہنچے۔				
18	جب میں کچھ غلط کروں تو مجھے پچھتاوے کا احساس نہیں ہوتا۔				
19	میں بہت جذباتی اور کھل کر اظہار کرنے والی/والا ہوں۔				
20	میں چیزیں بہتر کرنے کے لئے وقت صرف کرنا پسند نہیں کرتی/کرتا۔				
21	دوسروں کے احساسات میرے لیے غیر اہم ہیں۔				
22	میں دوسروں سے اپنے احساسات پوشیدہ رکھتی/رکھتا ہوں۔				
23	میں جو بھی کرتی/کرتا ہوں، سخت محنت سے کرتی/کرتا ہوں۔				
24	میں وہ کام کرتی/کرتا ہوں، جس سے لوگ اچھا محسوس کریں۔				
